# The effects of prolonged pacifier use on language development in infants and toddlers

**DOI:** 10.3389/fpsyg.2024.1349323

**Published:** 2024-02-20

**Authors:** Alexandros K. Kanellopoulos, Sarah E. Costello

**Affiliations:** Brain Health Department, Nestlé Institute of Health Sciences, Société des Produits Nestlé SA, Vers-Chez-les-Blanc, Lausanne, Switzerland

**Keywords:** speech, soother, cognition, socio-emotional, child development, language, pacifier, brain

## Abstract

Pacifiers are a common soothing tool used by parents to calm and comfort infants and toddlers. While pacifiers can provide temporary relief, there is growing concern about the potential long-term effects of prolonged pacifier use on language and cognitive development. Previous studies have suggested that prolonged use of pacifiers may have negative consequences on language outcomes in infants and toddlers, especially during the first few years of life known to be a critical period for language development. Previous studies have shown that children who use pacifiers extensively have smaller vocabulary sizes at 1 and 2 years of age which can have subsequent effects on socioemotional. In addition, significant association between greater frequency of daytime pacifier use and worsening of cognitive outcomes was shown. Furthermore, research has shown a strong dose–response association between intense pacifier use up to 4 years of age and lower IQ at 6 years. Recently, the importance of oral motor movements and sensorimotor production for speech perception in infants as young as 6 months has been highlighted, raising important questions on the effect of oral motor movement restrictions at an early age. Together, these findings raise concern about the potential long-term effects of prolonged pacifier use on language and cognitive development at a critical time in child development. However, it is still debatable within the scientific field the potential relationship between pacifier use and language development in early life most likely due to the complexity of studying child development. This mini review aims to provide valuable insights for parents, caregivers, and healthcare professionals in making informed decisions and understand regarding pacifier use for infants and toddlers.

## 1 Introduction

Language development is a crucial aspect of child development, with the first few years of life being a critical period for this skill acquisition (Barca, 2021). During this time, infants rapidly develop the ability to understand and produce speech, with various developing brain areas involved in the process. A controversial yet common practice during infancy that often extends to the toddler years, is the use of pacifiers, also known as soothers and dummies. While using pacifiers for non-nutritive sucking (NNS) provides positive comforting effects, several organizations and healthcare professionals share concern over their usage (Ponti, 2003).

Pacifiers, which are typically made of silicone or rubber, are the most common tool used by parents to soothe infants and young children, with studies reporting usage rates of up 84% (Ponti, 2003; Barca et al., 2017). While pacifiers can provide comfort and reduce the risk of sudden infant death syndrome, negative reports associate them with a reduction in breastfeeding duration (Buccini et al., 2017), increased frequency of otitis media (Salah et al., 2013) and dental problems, including the growth and development of a child’s jaw, baby teeth, and oral muscles (Gederi et al., 2013) and dental malocclusion, including open bite, overbite and crossbite (Medeiros et al., 2018). In addition to these more well-established concerns, more recent work suggests pacifier use may also interfere with speech development, igniting debate over the potential impacts on language at this critical time of development (Adair, 2003).

Considering these new concerns, this mini review aims to provide an update on the research examining the impact of prolonged pacifier use on speech and language development and to offer a comprehensive understanding of the potential benefits and risks. In general, the use of pacifiers is a subject of controversy, with conflicting recommendations in published literature. While the evidence is far from being conclusive (Nelson, 2012; Strutt et al., 2021), current results suggest that pacifier use may have negative implications for speech and language development, especially after 3 years of age (Adair, 2003; Barca et al., 2017, 2020; Barca, 2021). Considering this, it is important for parents and caregivers to be aware of the potential risks associated with prolonged pacifier use and to monitor their child’s use accordingly.

## 2 The effects of pacifier use on language development in infants and toddlers

Pacifier use has been linked to negative effects on speech and language development in infants and toddlers. Prolonged use of pacifiers can lead to raised or indented palates, which can result in an oral cavity that is too large for normal articulation (Choi et al., 2019). According to research, intense use of pacifiers, defined as use over several hours during the day, can have a detrimental effect on speech and language development, especially for children past 2–3 years of age (Giugliani et al., 2021; Strutt et al., 2021). A recent study found that using the pacifier beyond 3 years of age affects abstract word processing later in life (Barca et al., 2020). In support of this, Strutt and colleagues found that intense pacifier use may start to have clinical implications for oral motor and language development (Strutt et al., 2021). However, this study found that most speech outcomes in children were not significantly associated with pacifier use, except for an increased frequency of atypical errors linked to daytime use.

In addition to the negative effects on speech and language development, prolonged pacifier use can also lead to delayed oral motor development (Adair, 2003; Barca et al., 2017). This is of significance when it is considered that lower levels of oral motor skills correspond with lower levels of language (Alcock, 2006). Indeed, oral motor skills have been shown to be associated with language production at 21 months and 3 years of age (Alcock and Connor, 2021) and grammatical vocabulary at 24 months (Alcock and Gordon, 2002). Thus, it appears pacifiers reduce the opportunity for infants to practice oral motor movements, such as tongue and lip movements, which are critical for speech development and production. This is of particular importance as theories of language acquisition have typically assumed an infant’s perceptual capabilities influence the development of speech production. However, a recent study by Bruderer et al. shows for the first time, that sensorimotor production also influences speech perception (Bruderer et al., 2015). More specifically, the study found articulatory configurations affected the way infants perceived speech, illustrating how speech production system shapes speech from early life (Bruderer et al., 2015). These findings importantly implicate oral motor movements to be more significant to speech perception and language acquisition than previously believed, and highlight the significant effect restricting oral motor movements, via pacifiers for example, could have on speech and language in infants and toddlers.

Prolonged use of pacifiers has also been shown to alter facial expressions in children which can affect the development of their emotional competence and communication skills (Barca et al., 2017). Thus, this reduced opportunity for language exposure and practice can further contribute to delayed language development in infants, with early language delays being associated with poorer socio-emotional functioning (Tsao et al., 2004; Kuhl et al., 2005; Rajalin et al., 2021), reduced social interaction (Gertner et al., 1994), poorer literacy skills in later childhood (Preston et al., 2010) and academic achievement (Bleses et al., 2016). Furthermore, vocabulary at 24 and 28 months of age is predictive of later school readiness (Hammer et al., 2017), with attainment of early language milestones predicting later academic success (Walker et al., 1994; Im-Bolter et al., 2013). Therefore, it is important to provide infants and toddlers the best opportunities to progress language skills at an early age.

Prolonged pacifier use has been found to increase the risk of ear infections, which have been linked to speech and language development delays (Nelson, 2012). Gradually reducing pacifier use until the child no longer needs it could help mitigate some of the potential negative effects on development (Burr et al., 2021). It is important for parents and caregivers to be aware of the potential negative effects of pacifier use on speech and language development and to promote healthy oral motor development in infants through alternative means, such as engaging in face-to-face communication, singing, and reading aloud to infants.

## 3 Mediating factors that impact the effects of pacifier use on language development

The frequency (how often per day) and duration (in number of months) of pacifier use play a significant role in their impact on language development (Barca et al., 2017; Strutt et al., 2021). Studies suggest that prolonged use of pacifiers beyond the age of 3 can negatively impact speech development (Dogramaci and Rossi-Fedele, 2016). For example, Barca et al. (2017) demonstrated that among children who overused pacifiers for more than 3 years, distinct patterns in conceptual relations emerged, with less clear distinctions between certain types of concepts and a tendency to rely less on personal experiences and more on exemplifications and functional relations in their definitions. However, the main effect observed in this study indicates that children demonstrated higher accuracy in defining concrete and abstract emotional concepts compared to abstract not-emotional concepts, irrespective of pacifier use. Moreover, excessive use of pacifiers leading to prolonged sucking can affect the development of the oral muscles required for speech production (Bruderer et al., 2015). Parents should therefore monitor the frequency and duration of pacifier use to minimize their potential impact on language development.

The age at which pacifiers are introduced can also affect on language development (Adair, 2003; Fernandez, 2016). While the American Academy of Pediatrics recommends introducing pacifiers after breastfeeding, which is well-established, some experts suggest waiting until at least six months of age (Adair, 2003), with the suggestion that the introduction of pacifiers too early can interfere with the development of oral muscles and negatively impact speech development (Fernandez, 2016). Genetics and individual differences may also play a role in the impact of pacifier use on language development (Ponti, 2003; Cinar, 2004; Medeiros et al., 2018). Indeed some studies suggest that prolonged use of pacifiers can lead to dental malocclusion and subsequent speech difficulties (Cinar, 2004; Medeiros et al., 2018), while others suggest that these problems only arise with prolonged or inappropriate use (Ponti, 2003). Additionally, individual differences in oral motor development and speech abilities may influence the impact of pacifier use on language development (Barca et al., 2017).

A recent study moved beyond the findings of momentary effects of experimentally induced “impairment” in articulators’ movement on speech perception. The findings suggested that from 12 months of age constraints on infant’s speech articulators via pacifier use, may be negatively associated with word comprehension and production (Muñoz et al., 2021). Given this evidence, parents should consider individual factors when deciding if, and when to use pacifiers, while monitoring their child’s language development to ensure it is not being negatively impacted.

## 4 Strategies for minimizing the negative effects of pacifier use on language development

Limiting pacifier use is an essential strategy for minimizing potential negative effects on language development (Barca et al., 2017). While pacifiers can provide comfort and soothe infants, prolonged use can lead to changes in the mouth and jaw, potentially affecting speech and language development (Burr et al., 2021). Parents can limit pacifier use by gradually reducing their frequency and duration of use, especially as the child gets older (Gederi et al., 2013). It is also essential to avoid using pacifiers as a substitute for other forms of comfort and attention, such as holding and cuddling (Strutt et al., 2021). By limiting pacifier use, parents can promote healthy oral development and minimize potential negative effects on language development.

Encouraging alternative forms of soothing is another effective strategy for minimizing negative effects of pacifier use on language development (Cinar, 2004). Parents can encourage their infants to self-soothe by using other methods, such as gentle rocking, singing, and swaddling (Adair, 2003). Additionally, parents can provide alternative comfort objects, such as soft blankets or stuffed animals, to help their infants feel secure and calm (Cinar, 2004). By promoting alternative forms of soothing, parents can reduce their reliance on pacifiers, potentially minimizing the negative impact on language development.

Promoting language exposure and practice is a crucial strategy for supporting language development independent of pacifier use (Abu-Zhaya et al., 2023). Parents can promote language development by engaging in frequent conversation with their children, reading books together, having family mealtimes and singing songs (Cinar, 2004). It’s important to provide a language-rich environment, with exposure to a variety of sounds, words, and experiences. By promoting language exposure and practice, parents can help their infants develop strong language skills, potentially minimizing the negative effects of pacifier use on language development.

## 5 Benefits and risks of long-term pacifier usage on cognitive development

Non-nutritive sucking involves sucking on a pacifier, thumb, or other objects without receiving any food or nutrients and is thought to be an essential part of early development as it provides sensory stimulation and promotes oral motor skill development. Furthermore, research has shown that NNS can have a positive impact on brain development, with studies suggesting that it can accelerate the attainment of developmental milestones. By engaging in NNS, infants may be able to strengthen the neural connections involved in language development and other cognitive functions.

Using pacifiers too often, however, may have negative effects on cognitive development. For example, intense pacifier usage up to 4 years of age has been significantly associated with lower IQ at age 6 (Lehtonen et al., 2016). This correlation follows a dose–response gradient, meaning that the longer the duration of pacifier use, the greater the IQ deficit (Lehtonen et al., 2016). While the exact mechanisms by which pacifier use reduces IQ are still unclear, one hypothesis suggests that children who use a pacifier, especially intensively, may experience less stimulation (Lehtonen et al., 2016). In addition, research has shown that children who used a pacifier all day had a lower IQ compared to those who never used it (Lehtonen et al., 2016). This inverse association between pacifier use and IQ is further supported by the findings that nutritional sucking has a direct effect on the cortical activity of newborns, causing a significant reduction in brain power and reduced alertness, as assessed via electroencephalography. However, this response declines during the neonatal period and is absent at 12 weeks. In 24-week-old infants, nutritional sucking is accompanied by an increase in rhythmic theta neurophysiological activity, but not directly to alertness change (Friederici, 2011). These findings suggest a developmental relationship between nursing and infant brain function with plausible affective and cognitive implications. Despite this strong correlation, further research is required to clarify the underlying mechanisms (Lehtonen et al., 2016).

## 6 Conclusion and future research directions

While the evidence is not conclusive, an increasing body of work suggests that the prolonged use of pacifiers may have unintended consequences on children’s development and health. More specifically, research on the effects of long-term pacifier use on language development suggests that prolonged use of these devices may have negative consequences on speech, language acquisition, and cognitive development (Adair, 2003; Barca et al., 2017), especially in toddlers. The negative effects include delayed oral motor development and reduced opportunities for language exposure and practice alongside potential implications for socio-emotional and cognitive development. The frequency (how many times per day), intensity (how long per day), and duration (in months) of pacifier use underlie the definition of prolonged pacifier use and are related to type and extent of the above risks (Nelson, 2012; Muñoz et al., 2021). The findings suggest that prolonged pacifier use during the day may have a subtle impact on speech, but professionals should be cautious given the small evidence base so far. In general, the pacifier use at older ages, beyond 2 years old, correlates stronger (and negatively) with vocabulary size than more precocious pacifier use (Muñoz et al., 2021). Overall, there is a gap in research on the speech effects of pacifier use, which has been characterized by small sample sizes and insufficient measures in past studies (Strutt et al., 2021).

As several studies do not show an association between the pacifier use and language development, this lack of a definitive consensus can be due to several factors such as socioeconomic status (SES), parental interaction, incidence of ear infections, and genetic predispositions. Several studies have consistently highlighted a robust association between socioeconomic status and language development. Children from higher SES backgrounds tend to be exposed to linguistically enriched environments, positively shaping their language skills (Hoff, 2003). Hoff’s (2003) study specifically underscores the influence of maternal speech on early vocabulary development in children, emphasizing the specificity of environmental influence. On this notion of enriched environment, the quality and quantity of parent–child interaction also play a crucial role in language acquisition. Therefore, responsive and stimulating interactions enhance language development in children (Hart and Risley, 1995). In addition, genetic factors contribute significantly to language development, influencing key aspects such as phonological processing and language learning abilities (Bishop, 2002). Collectively, children exhibit significant individual variability in their development, including language acquisition. Factors such as genetic predisposition, cognitive abilities, and socio-economic environment can all play a role. This makes it challenging to establish a direct and uniform correlation between pacifier use and language development (Muñoz et al., 2021).

Therefore, it is well appreciated that child development is influenced by a myriad of variables, and isolating the impact of a single factor, such as pacifier use, can be challenging. Researchers often attempt to control for various confounding variables, but the dynamic nature of child development makes it challenging to attribute outcomes solely to one factor. In combination with the temporal aspects as child development is a dynamic process, during which the outcomes can change over time. What might appear as a correlation between pacifier use and language development at one age may not persist or may manifest differently as the child grows. Longitudinal studies that track development over time are essential but can be resource-intensive.

A noteworthy consideration in language development is the impact of recurrent ear infections, which have been correlated with temporary hearing loss affecting a child’s ability to hear and learn language (Paradise et al., 2003). The findings suggest a correlation between early ear infections and subsequent language delays, underscoring the importance of auditory capacity in language acquisition.

Research findings can vary based on the design and methodology of studies. Some studies may have limitations, such as small sample sizes, differences in participant demographics, or varying definitions of pacifier use (Strutt et al., 2021). These variations can lead to conflicting results and difficulties in drawing broad conclusions.

Understanding the collective impact of these factors necessitates comprehensive research that considers their interactions within the unique circumstances of individual children. It is imperative to acknowledge the dynamic nature of the field of language development, with ongoing research poised to provide additional insights into the intricate interrelationships among these factors. As our understanding evolves, continued exploration of these influences will deepen our comprehension of the complexities inherent in language development. Strategies for minimizing the negative effects of pacifier use on language development include limiting use, encouraging alternative forms of soothing, and promoting language exposure and practice. These findings have important implications for parents, caregivers and healthcare professionals who should be aware of these potential effects and take steps to provide an optimal environment for the promotion of healthy language development in infants and toddlers. Future research should continue to explore the long-term effects of pacifier use on children’s language development. Specifically, research should investigate the optimal duration and frequency of pacifier use, as well as the potential benefits and risks associated with different types of pacifiers (Bruderer et al., 2015; Barca, 2021). Furthermore, it is yet to be understood what the underlying mechanisms are, through which pacifier use may impact language development and dental health, including the effects on oral motor skills and muscle development (Barca et al., 2017; Strutt et al., 2021). By continuing to investigate these questions, we can better understand the potential risks and benefits of pacifiers and provide evidence-based recommendations for health care and educational communities.

## Author contributions

AK: Conceptualization, Writing – original draft, Writing – review & editing. SC: Writing – review & editing, Conceptualization.

## References

[ref1] Abu-Zhaya R. Goffman L. Brosseau-Lapre F. Roepke E. Seidl A. (2023). The effect of somatosensory input on word recognition in typical children and those with speech sound disorder. J. Speech Lang. Hear. Res. 66, 84–97. doi: 10.1044/2022_JSLHR-22-00226 36603544 PMC10023182

[ref2] Adair S. M. (2003). Pacifier use in children: a review of recent literature. Pediatr. Dent. 25, 449–458. PMID: 14649608

[ref3] Alcock K. J. (2006). The development of oral motor control and language. Down Synd. Res. Pract. 11, 1–8. doi: 10.3104/reports.310 17048804

[ref4] Alcock K. Connor S. (2021). Oral motor and gesture abilities independently associated with preschool language skill: longitudinal and concurrent relationships at 21 months and 3–4 years. J. Speech Lang. Hear. Res. 64, 1944–1963. doi: 10.1044/2021_JSLHR-19-00377, PMID: 33979210

[ref5] Alcock K Gordon E. Oral motor and language development. Euresco Conference Series-Brain and Cognition in Human Infants (2002).

[ref6] Barca L. (2021). Toward a speech-motor account of the effect of age of pacifier withdrawal. J. Commun. Disord. 90:106085. doi: 10.1016/j.jcomdis.2021.106085, PMID: 33550069

[ref7] Barca L. Mazzuca C. Borghi A. M. (2017). Pacifier overuse and conceptual relations of abstract and emotional concepts. Front. Psychol. 8:2014. doi: 10.3389/fpsyg.2017.02014 29250003 PMC5717369

[ref8] Barca L. Mazzuca C. Borghi A. M. (2020). Overusing the pacifier during infancy sets a footprint on abstract words processing. J. Child Lang. 47, 1084–1099. doi: 10.1017/S0305000920000070, PMID: 32345380

[ref9] Bishop D. V. (2002). The role of genes in the etiology of specific language impairment. J. Commun. Disord. 35, 311–328. doi: 10.1016/S0021-9924(02)00087-4 12160351

[ref10] Bleses D. Makransky G. Dale P. S. Højen A. Ari B. A. (2016). Early productive vocabulary predicts academic achievement 10 years later. Appl. Psycholinguist. 37, 1461–1476. doi: 10.1017/S0142716416000060

[ref11] Bruderer A. G. Danielson D. K. Kandhadai P. Werker J. F. (2015). Sensorimotor influences on speech perception in infancy. Proc. Natl. Acad. Sci. U.S.A. 112, 13531–13536. doi: 10.1073/pnas.1508631112, PMID: 26460030 PMC4640749

[ref12] Buccini G. D. S. Pérez-Escamilla R. Paulino L. M. Araujo C. L. Venancio S. I. (2017). Pacifier use and interruption of exclusive breastfeeding: systematic review and meta-analysis. Matern. Child Nutr. 13:e12384. doi: 10.1111/mcn.12384, PMID: 27863027 PMC6866034

[ref13] Burr S. Harding S. Wren Y. Deave T. (2021). The relationship between feeding and non-nutritive sucking behaviours and speech sound development: a systematic review. Folia Phoniatr. Logop. 73, 75–88. doi: 10.1159/000505266, PMID: 32040950

[ref14] Choi D. Bruderer A. G. Werker J. F. (2019). Sensorimotor influences on speech perception in pre-babbling infants: replication and extension of Bruderer et al. (2015). Psychon. Bull. Rev. 26, 1388–1399. doi: 10.3758/s13423-019-01601-0, PMID: 31037603 PMC6711806

[ref15] Cinar D. N. (2004). The advantages and disadvantages of pacifier use. Contemp. Nurse 17, 109–112. doi: 10.5172/conu.17.1-2.109, PMID: 17929742

[ref16] Dogramaci E. J. Rossi-Fedele G. (2016). Establishing the association between nonnutritive sucking behavior and malocclusions: a systematic review and meta-analysis. J. Am. Dent. Assoc. 147, 926–934.e6. doi: 10.1016/j.adaj.2016.08.018, PMID: 27692622

[ref17] Fernandez S. (2016). Oral health basics—what every pediatrician should know. Pediatr. Ann. 45, e379–e381. doi: 10.3928/19382359-20161018-02 27841918

[ref18] Friederici A. D. (2011). The brain basis of language processing: from structure to function. Physiol. Rev. 91, 1357–1392. doi: 10.1152/physrev.00006.2011 22013214

[ref19] Gederi A. Coomaraswamy K. Turner P. J. (2013). Pacifiers: a review of risks vs benefits. Dent. Update 40, 92–101. doi: 10.12968/denu.2013.40.2.92 23600033

[ref20] Gertner B. L. Rice M. L. Hadley P. A. (1994). Influence of communicative competence on peer preferences in a preschool classroom. J. Speech Lang. Hear. Res. 37, 913–923. doi: 10.1044/jshr.3704.913 7967575

[ref21] Giugliani E. R. J. Gomes E. Santos I. S. Matijasevich A. Camargo-Figuera F. A. Barros A. J. D. (2021). All day-long pacifier use and intelligence quotient in childhood: a birth cohort study. Paediatr. Perinat. Epidemiol. 35, 511–518. doi: 10.1111/ppe.12752, PMID: 33570810

[ref22] Hammer C. S. Morgan P. Farkas G. Hillemeier M. Bitetti D. Maczuga S. (2017). Late talkers: a population-based study of risk factors and school readiness consequences. J. Speech Lang. Hear. Res. 60, 607–626. doi: 10.1044/2016_JSLHR-L-15-0417, PMID: 28257586 PMC5962923

[ref23] Hart B. Risley T. R. (1995). Meaningful differences in the everyday experience of young American children Baltimore, USA: Paul H Brookes Publishing.

[ref24] Hoff E. (2003). The specificity of environmental influence: socioeconomic status affects early vocabulary development via maternal speech. Child Dev. 74, 1368–1378. doi: 10.1111/1467-8624.00612, PMID: 14552403

[ref25] Im-Bolter N. Yaghoub Zadeh Z. Ling D. (2013). Early parenting beliefs and academic achievement: the mediating role of language. Early Child Dev. Care 183, 1811–1826. doi: 10.1080/03004430.2012.755964

[ref26] Kuhl P. K. Conboy B. T. Padden D. Nelson T. Pruitt J. (2005). Early speech perception and later language development: implications for the" critical period". Lang. Learn. Dev. 1, 237–264. doi: 10.1207/s15473341lld0103&4_2

[ref27] Lehtonen J. Valkonen-Korhonen M. Georgiadis S. Tarvainen M. P. Lappi H. Niskanen J. P.. (2016). Nutritive sucking induces age-specific EEG-changes in 0-24 week-old infants. Infant Behav. Dev. 45, 98–108. doi: 10.1016/j.infbeh.2016.10.005, PMID: 27792918

[ref28] Medeiros R. Ximenes M. Massignan C. Flores-Mir C. Vieira R. Porporatti A. L.. (2018). Malocclusion prevention through the usage of an orthodontic pacifier compared to a conventional pacifier: a systematic review. Eur. Arch. Paediatr. Dent. 19, 287–295. doi: 10.1007/s40368-018-0359-3, PMID: 30054865

[ref29] Muñoz L. E. Kartushina N. Mayor J. (2021). Sustained pacifier use is associated with smaller vocabulary sizes at 1 and 2 years of age. Psy ArXiv. doi: 10.31234/osf.io/guek6 38270235

[ref30] Nelson A. M. (2012). A comprehensive review of evidence and current recommendations related to pacifier usage. J. Pediatr. Nurs. 27, 690–699. doi: 10.1016/j.pedn.2012.01.004, PMID: 22342261

[ref31] Paradise J. L. Dollaghan C. A. Campbell T. F. Feldman H. M. Bernard B. S. Colborn D. K.. (2003). Otitis media and tympanostomy tube insertion during the first three years of life: developmental outcomes at the age of four years. Pediatrics 112, 265–277. doi: 10.1542/peds.112.2.265, PMID: 12897272

[ref32] Ponti M. (2003). Recommendations for the use of pacifiers. Paediatr. Child Health 8, 515–519. doi: 10.1093/pch/8.8.515, PMID: 20019941 PMC2791559

[ref33] Preston J. L. Frost S. J. Mencl W. E. Fulbright R. K. Landi N. Grigorenko E.. (2010). Early and late talkers: school-age language, literacy and neurolinguistic differences. Brain 133, 2185–2195. doi: 10.1093/brain/awq163, PMID: 20826428 PMC3139938

[ref34] Rajalin S. Pihlaja P. Carter A. S. C. Rautakoski P. (2021). Associations between social emotional and language domains in toddlerhood–the steps study. J. Child Lang. Acquis. Dev. 9, 223–248.

[ref35] Salah M. Abdel-Aziz M. Al-Farok A. Jebrini A. (2013). Recurrent acute otitis media in infants: analysis of risk factors. Int. J. Pediatr. Otorhinolaryngol. 77, 1665–1669. doi: 10.1016/j.ijporl.2013.07.022, PMID: 23953241

[ref36] Strutt C. Khattab G. Willoughby J. (2021). Does the duration and frequency of dummy (pacifier) use affect the development of speech? Int. J. Lang. Commun. Disord. 56, 512–527. doi: 10.1111/1460-6984.12605, PMID: 33939239

[ref37] Tsao F. M. Liu H. M. Kuhl P. K. (2004). Speech perception in infancy predicts language development in the second year of life: a longitudinal study. Child Dev. 75, 1067–1084. doi: 10.1111/j.1467-8624.2004.00726.x 15260865

[ref38] Walker D. Greenwood C. Hart B. Carta J. (1994). Prediction of school outcomes based on early language production and socioeconomic factors. Child Dev. 65, 606–621. doi: 10.2307/1131404 8013242

